# KRAS, NRAS, BRAF signatures, and MMR status in colorectal cancer patients in North China

**DOI:** 10.1097/MD.0000000000033115

**Published:** 2023-03-03

**Authors:** Shen-Yi Lian, Lu-Xin Tan, Xin-Zhi Liu, Lu-Jing Yang, Ning-Ning Li, Qing Feng, Ping Wang, Yue Wang, Dong-Bo Qiao, Li-Xin Zhou, Ting-Ting Sun, Lin Wang, Ai-Wen Wu, Zhong-Wu Li

**Affiliations:** a Department of Pathology, Key Laboratory of Carcinogenesis and Translational Research (Ministry of Education), Peking University Cancer Hospital & Institute, Beijing, China; b Department of Colorectal Surgery, Key Laboratory of Carcinogenesis and Translational Research (Ministry of Education), Peking University Cancer Hospital & Institute, Beijing, China.

**Keywords:** BRAF, colorectal cancer, KRAS, microsatellite instability, NRAS, prognosis

## Abstract

We assessed the clinicopathological features and prognostic values of KRAS, NRAS, BRAF, and DNA mismatch repair status in colorectal cancer (CRC) to provide real-world data in developing countries. We enrolled 369 CRC patients and analyzed the correlation between RAS/BRAF mutation, mismatch repair status with clinicopathological features, and their prognostic roles. The mutation frequencies of KRAS, NRAS, and BRAF were 41.7%, 1.6%, and 3.8%, respectively. KRAS mutations and deficient mismatch repair (dMMR) status were associated with right-sided tumors, aggressive biological behaviors, and poor differentiation. BRAF (V600E) mutations are associated with well-differentiated and lymphovascular invasion. The dMMR status predominated in young and middle-aged patients and tumor node metastasis stage II patients. dMMR status predicted longer overall survival in all CRC patients. KRAS mutations indicated inferior overall survival in patients with CRC stage IV. Our study showed that KRAS mutations and dMMR status could be applied to CRC patients with different clinicopathological features.

## 1. Introduction

As one of the most common cancers, genetics and biomarkers of colorectal cancer (CRC) have been extensively researched. When discussing the specific molecular mechanisms underlying the development and progression of CRC, we always think of adenomatous polyposis coli, deleted in colon cancer, the RAS-RAF-MAPK signaling pathway, and microsatellite status. Mutations in proto-oncogenes, tumor suppressor genes, and genes related to DNA mismatch repair mechanisms play key roles in CRC development.^[1,2]^ However, in clinical practice we are more concerned with the molecular changes that occur during the initiation, progression, and metastasis of CRC, which have been proven to be useful in predicting behavior and prognosis and guiding treatment. With improvements in medical treatment and the development of precision medicine, we should consider the clinical significance of genetic variances, such as microsatellite instability (MSI) as well as mutations in KRAS, NRAS, and BRAF before planning treatment modalities.^[3,4]^

Microsatellites are repetitive sequences of dozens of nucleotides in the human genome that contain units composed of a few nucleotides. These sequences are particularly prone to accumulate mutations, insertions, and deletions in DNA coding regions that lead to protein truncations.^[5]^ Fortunately, a highly conservative and effective monitoring system, mismatch repair (MMR), has always been prepared to correct the errors that occur in microsatellites. MLH1, MLH2, MLH6, and PMS2 are the main proteins in this system. They usually interact as heterodimers (MLH1 couples with PMS2 while MLH2 associates with PMS2), and the lack of expression of one or more of these proteins causes deficient MMR (dMMR); otherwise, MMR is considered proficient (pMMR).^[6]^ Therefore, what we propose for microsatellite instability (MSI) is that the mutations that occur in genes responsible for surveillance and modification lead to an accumulation of errors in DNA.^[7]^ The opposite is considered microsatellite stability (MSS). MSI is present in approximately 15% of all CRC cases. However, it is significant for guiding clinical treatments and can be used not only for screening Lynch syndrome, which is an autosomal dominant disease that accounts for 2 to 3% of CRC cases but also for guiding immune checkpoint therapy after receiving regulatory approval in 2017.^[8]^

Activation of the RAS-RAF-MEK-ERK signaling pathway is the basis of tumorigenesis, which drives cell proliferation, differentiation, and migration, and occurs in multiple tumor types. This most frequently manifests as mutations in the KRAS, NRAS, and BRAF genes.^[9,10]^ RAS mutations have been found in one-third of all cancers, of which KRAS mutations are the most prevalent (>80%) whereas mutations in NRAS (approximately 10%) and HRAS (<5%) are relatively rare. BRAF mutations have been detected in 7 to 8% of cancers, and the V600E mutation accounts for >90% of BRAF mutations.^[11]^ KRAS mutations are negative prognostic factors for the efficacy of anti-epidermal growth factor receptor therapy for advanced CRC, which means patients benefit little from anti-epidermal growth factor receptor therapy.^[12,13]^

We designed a retrospective study to explore the potential correlations among KRAS/NRAS/BRAF mutations, MMR status, clinicopathological features, and prognosis. We believed that these results would provide a basis for the molecular epidemiology of Chinese CRC patients and future clinical trials in China.

## 2. Materials and methods

### 2.1. Patients

This analysis was based on a database of patients at Beijing Cancer Hospital from January 2014 to December 2018; 629 patients had intact CRC-related driver gene mutations (KRAS, NRAS, and BRAF) and MMR status. The following exclusion criteria were applied: patients who underwent neoadjuvant therapy before surgery, insufficient clinical pathological characteristics, unavailability of surgically excised specimens, and no paraffin-embedded specimens for pathology. All patients were diagnosed with CRC by 2 independent pathologists. A total of 369 patients were included in the analysis; however, 10 patients were excluded from the survival analysis because of the lack of follow-up.

All human data analyses conducted in this study were approved by the Ethics Committee of the Peking University Cancer Hospital. Written informed consent was obtained from all the participants. This study was conducted in accordance with the principles of the Declaration of Helsinki.

### 2.2. DNA extraction and mutation analysis

Tumor DNA was extracted from formalin-fixed, paraffin-embedded tissues containing >20% tumor cells using a QIAamp DNA Mini Kit (Qiagen). KRAS (Human KRAS Gene Mutation Detection Kit [Fluorescent PCR], Lot: TB001-B3), NRAS (Human NRAS Gene Mutation Detection Kit [Fluorescent PCR], Lot: TB108-B3), BRAF (Human BRAF Gene Mutation Detection Kit [Fluorescent PCR], Lot: TB004-B3) gene mutations, were detected centrally at the Department of Pathology at Beijing Cancer Hospital. These included KRAS/NRAS exon 2/3 hotspot mutations and BRAF V600E mutation.

### 2.3. Microsatellite status determination

MMR status was detected by immunohistochemistry (IHC) for MLH1, MSH2, MSH6, and PMS2. Any positive reaction in the cancer cell nucleus was considered complete expression (+, normal) according to CAP Biomarker Reporting Protocols (Colon and Rectum Biomarker Reporting, version 1.3.0.0). Applying the principle of specific binding of antibodies and antigens, the absence of the main proteins was tested using a Leica BOND-III fully automated IHC and In Situ Hybridization Staining System (Leica Biosystems, Buffalo Grove, IL). All the results were confirmed by at least 2 experienced pathologists.

### 2.4. Statistical analysis

All statistical analyses were performed using IBM SPSS Statistics (version 26.0; IBM Corporation, Armonk, NY). The Kolmogorov–Smirnov test was used to verify the assumptions of normal distribution. The chi-square test or Fisher exact test was used to compare categorical variables. Overall survival (OS) was defined as the period between the first surgery and death from any cause. Disease-free survival (DFS) was defined as the time from the date of initial surgery to the date of death for any cause or the first local recurrence or metastasis. Cox proportional hazard models were used to analyze the prognostic predictors. Univariate and multivariate analyses were performed for predictors. Log-rank tests were used to identify the relationship between OS and predictors, and Kaplan–Meier survival curves were used to present the results. Statistical significance was set at *P* < .05.

## 3. Results

### 3.1. The clinical characteristics of 369 patients

The average age of the patients with CRC in our cohort was 61 years. The incidence of CRC was higher in men (67.2%, 248/369) than in women (32.8%, 121/369). Of the 369 patients in our cohort, 149 (40.4%) had tumors in the rectum, 131 (35.5%) in the left colon (splenic flexure and sigmoid colon), and 87 (23.6%) in the right colon (cecum, hepatic flexure, and transverse colon). Adenocarcinomas (91.1%, 336/369) were the most common histological type, and 287 (77.8%) were moderately differentiated (World Health Organization Classification of Tumors of the Digestive System, 5th Edition). Lymphovascular invasion was observed in 104 (28.2%) samples and perineural invasion was observed in 93 (25.2%) samples.

In addition to the clinicopathological characteristics, we also detected the diver gene mutations and expression of mismatch repair proteins (MLH1, MSH2, MSH6, and PMS2). As listed in Table 1, 154 (41.7%) KRAS, 6 (1.6%) NRAS, and 14 (3.8%) BRAF (V600E) mutations were detected in our cohort, and dMMR status (IHC method) was found in 12.5% of patients (46/369). The detailed clinical characteristics are listed in Table 1.

**Table 1:** Clinical data of 369 patients.

Variables	n (%)
Age (year), 61.37 ± 11.39	
≤60	168 (45.5)
>60	201 (54.5)
Sex	
Male	248 (67.2)
Female	121 (32.8)
Tumor site	
Right	87 (23.6)
Left	131 (35.5)
Rectum	149 (40.4)
Multisite tumors	2 (0.5)
Histological type	
Adenocarcinoma	336 (91.1)
Mucinous carcinoma	25 (6.8)
Signet-ring cell carcinoma	7 (1.9)
Mixed adenoneuroendocrine carcinoma	1 (0.3)
Differentiation	
Well	12 (3.3)
Moderately	287 (77.8)
Poorly	37 (10.0)
Other/NA*	33 (8.9)
TNM stage	
TIS	3 (0.8)
I	56 (15.2)
II	149 (40.4)
III	125 (33.9)
IV	36 (9.8)
Lymphovascular invasion	
Positive	104 (28.2)
Negative	265 (71.8)
Perineural invasion	
Positive	93 (25.2)
Negative	276 (74.8)
KRAS	
WT	215 (58.3)
mut	154 (41.7)
NRAS	
WT	363 (98.4)
mut	6 (1.6)
BRAF	
WT	355 (96.2)
mut	14 (3.8)
MMR status	
pMMR	323 (87.5)
dMMR	46 (12.5)

dMMR = deficient mismatch repair, MMR = mismatch repair, mut = Mutation, pMMR = proficient mismatch repair, TIS = tumor in situ, WT = wild-type.

* include mucinous carcinoma, signet-ring cell carcinoma, and mixed adenoneuroendocrine carcinoma.

### 3.2. Association of KRAS, NRAS, and BRAF mutations with clinicopathologic features in CRC

Detailed information on KRAS, NRAS, and BRAF mutations is presented in Table 2. In total, 39.3% (145/369) of the KRAS mutations were in exon 2, and the mutation frequencies of KRAS codons 12 and 13 were 29.5% (109/369) and 9.8% (36/369), respectively. Only 2.4% (9/369) of KRAS Exon 3 codon 61 mutations were detected in our cohort. The majority of KRAS mutations were G12D or G12A in codon 12, which accounted for 16.8%, followed by G12V (7.6%), and G12S (2.7%). The frequency of the KRAS G12C mutation was 2.2% (8/369), which is consistent with published data. The frequency of NRAS mutations in exons 2 and 3 was both 0.8% (3/369). The frequency of BRAF (V600E) mutation was 3.8% (14/369) in our study. In general, the profiles of KRAS, NRAS, and BRAF mutations were in accordance with those reported in previous studies.

**Table 2:** Frequency and distribution of KRAS/NRAS/BRAF mutations.

Gene	Exon	Codon	Mutation	n (% of 369)
KRAS	2	12	G12S	10 (2.7)
			G12R	1 (0.3)
			G12C	8 (2.2)
			G12D/A	62 (16.8)
			G12V	28 (7.6)
		13	G13D	36 (9.8)
	3	61	Q61K, Q61L, Q61R, Q61P, Q61H	9 (2.4)
NRAS	2	12, 13	G12C, G12S, G13R	3 (0.8)
	3	61	Q61K or Q61L, Q61R or Q61H	3 (0.8)
BRAF	15	600	V600E	14 (3.8)

The associations between KRAS, NRAS, and BRAF mutations and clinicopathological features are listed in Table 3. KRAS mutations were only related to tumor location in 369 CRC patients. KRAS mutations were found in 62.1% of right-sided CRC patients, whereas KRAS mutations were found in 32.1% of left-sided and 37.6% of the rectum, respectively (*P* < .000). KRAS mutations were more prevalent in mucinous carcinoma than in adenocarcinoma or signet-ring cell carcinoma (72.0% vs 39.0% vs 57.1%, *P* = .006). There were 346 CRC patients with detailed differentiation information. KRAS mutations were found in 45.9% of CRC patients with poorly differentiated tumors, compared to 38.7 and 25.0% were found in moderately differentiated and well-differentiated (*P* = .004). Further, KRAS mutation was more prevalent in patients with negative lymphovascular invasion than in those with positive lymphovascular invasion (45.3% vs 32.7%, *P* = .037). However, KRAS mutations were not associated with age (*P* = .654), gender (*P* = .092), tumor node metastasis (TNM) stage (*P* = .172), or perineural invasion (*P* = .595).

**Table 3:** Association of KRAS NRAS, BRAF (V600E) mutations and MMR status and clinicopathologic features.

	KRAS		NRAS		BRAF	
	Mut (%)	*P*	Mut (%)	*P*	Mut (%)	*P*
Age (years)		*.654*		*.417*		*.587*
≤60	68 (40.5)		4 (2.4)		5 (3.0)	
>60	86 (42.8)		2 (1.0)		9 (4.5)	
Sex		*.092*		*.183*		*.779*
Male	96 (38.7)		6 (2.4)		9 (3.6)	
Female	58 (47.9)		0 (0)		5 (4.1)	
Tumor site		.000029		.445		.684
Right	54 (62.1)		1 (1.1)		5 (5.7)	
Left	42 (32.1)		4 (3.1)		5 (3.8)	
Rectum	56 (37.6)		1 (0.7)		4 (2.7)	
Multisite tumors	2 (100.0)		0 (0)		0 (0)	
Histological type		**.006**		.897		.956
Adenocarcinoma	131 (39.0)		6 (1.8)		13 (3.9)	
Mucinous carcinoma	18 (72.0)		0 (0)		1 (4.0)	
Signet-ring cell carcinoma	4 (57.1)		0 (0)		0 (0)	
MANEC*	1 (100.0)		0 (0)		0 (0)	
Differentiation		**.004**		.793		**.002**
Well	3 (25.0)		0 (0)		3 (25.0)	
Moderately	111 (38.7)		5 (1.7)		9 (3.1)	
Poorly	17 (45.9)		1 (2.7)		1 (2.7)	
Other/NA	23 (69.7)		0 (0)		1 (3.0)	
TNM stage		.172		*.939*		*.877*
TIS	3 (100.0)		0 (0)		0 (0)	
I	19 (33.9)		1 (1.8)		1 (1.8)	
II	67 (45.0)		3 (2.0)		6 (4.0)	
III	50 (40.0)		2 (1.6)		6 (4.8)	
IV	15 (41.7)		0 (0)		1 (2.8)	
Lymphovascular invasion		**.037**		.190		**.028**
Positive	34 (32.7)		0 (0)		8 (7.7)	
Negative	120 (45.3)		6 (2.3)		6 (2.3)	
Perineural invasion		.595		.779		.355
Positive	41 (44.1)		0 (0)		5 (5.4)	
Negative	113 (40.9)		6 (2.2)		9(3.3)	

* Mixed adenoneuroendocrine carcinoma.

BRAF (V600E) mutations were more associated with well-differentiated than poorly differentiated or moderately differentiated carcinomas (25.0% vs 3.1% vs 2.7%, *P* = .002). Patients with CRC with lymphovascular invasion had a higher mutation frequency of BRAF (V600E) than those without lymphovascular invasion (7.7% vs 2.3%, *P* = .028). The BRAF (V600E) mutation was not associated with age (*P* = .587), gender (*P* = .779), tumor site (*P* = .684), histological type (*P* = .956) TNM stage (*P* = .877), and perineural invasion (*P* = .355).

Different from KRAS and BRAF (V600E) mutation, none of the clinical characteristics, such as age, gender, tumor sites, histological type, differentiation, TNM stage, lymphovascular invasion, and perineural invasion, was associated with NRAS mutation. Above all, CRC patients with KRAS mutation or without BRAF mutation may have a better prognosis.

### 3.3. The prognostic impact of KRAS and BRAF mutation in CRC patients

To further confirm the prognostic role of driver gene mutation, we conducted Kaplan–Meier survival analysis in 363 CRC patients. Three cases of carcinoma in situ were excluded from this analysis. The median follow-up time was 49.3 months, and the OS time range was 1.1 to 92.8 months. In our study, of the 369 patients, only 163 had reliable DFS data.

**Figure 1:**
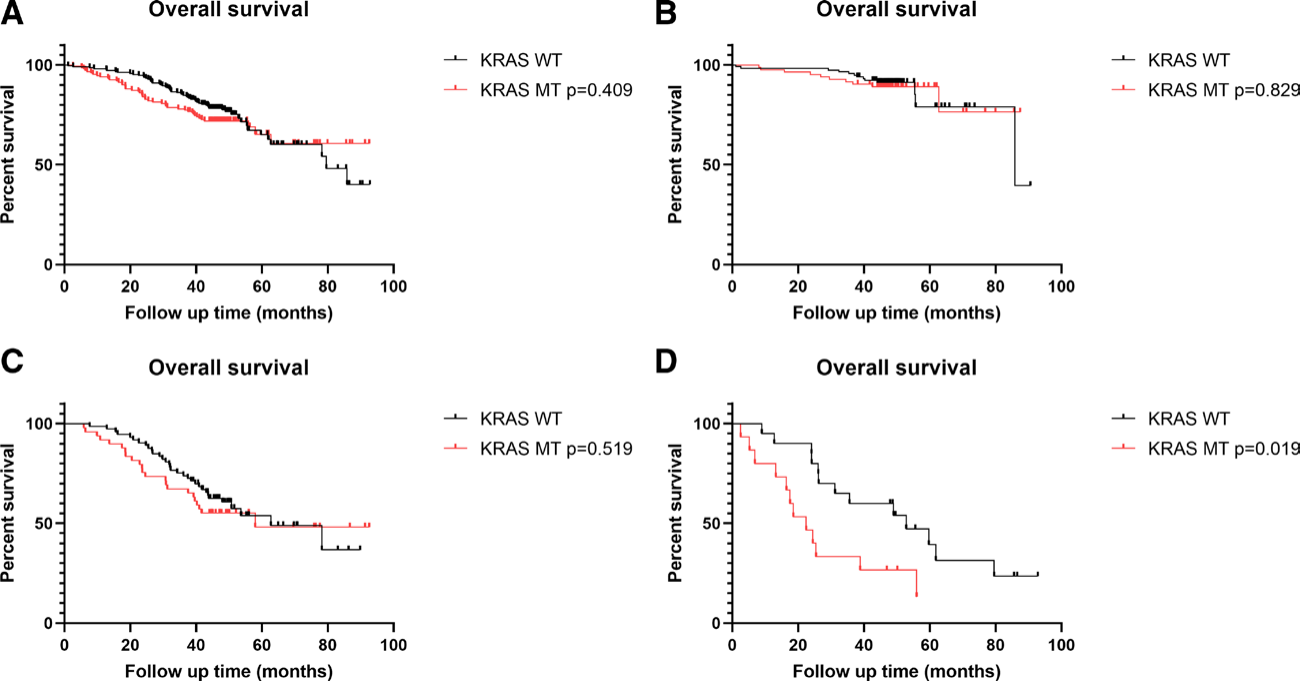
. KRAS mutation is associated with the prognosis in CRC patients at stage IV. (A) Comparing the overall survival in all the CRC patients (*P* = .829). (B) Comparing the overall survival in CRC patients at the early stage (I–II) (*P* = .829). (C) Comparing the overall survival in CRC patients at stage III (*P* = .519). (D) Comparing the overall survival in CRC patients at stage IV (*P* = .019). CRC = colorectal cancer.

The univariate and multivariate results of the general clinicopathological characteristics are listed in Supplementary Tables S1 and S2, Supplemental Digital Content, http://links.lww.com/MD/I559. Lymphovascular invasion (hazard ratio [HR] 2.585; 95% confidence interval [CI], 1.754–3.811; *P* < .000) and perineural invasion (HR 2.426; 95% CI, 1.636–3.597; *P* < .000) were both associated with poor prognosis in the univariate analysis but not in the multivariate analysis. Combined with the multivariate analysis results, age, TNM stage, histological type, and tumor differentiation were independent prognostic factors in patients with CRC. The univariate analysis of the driver gene mutation was listed in Table 4. There was no significant difference between the patients with driver gene mutations and those with wild-type in the univariate analysis. According to the univariate results, overall survival (OS) was not significantly different between the KRAS mutation and KRAS WT groups of all CRC patients or patients with the indicated stage (Fig. 1A–C). Patients with KRAS mutations had shorter survival times than the patients with wild-type KRAS in stage IV (*P* = .019) (Fig. 1D). There was also no association between KRAS mutations and DFS in our study (*P* = .224) (Supplementary Figure S1, Supplemental Digital Content, http://links.lww.com/MD/I560). In conclusion, KRAS mutation may serve as a prognostic factor in patients with late-stage CRC.

**Table 4:** Univariate analyses of prognostic impact of driver gene mutation.

Variables	Univariable analysis HR (95% CI)	*P*
KRAS mutation	1.179 (0.798–1.740)	.408
NRAS mutation	1.326 (0.326–5.385)	.693
BRAF mutation	1.608 (0.654–3.959)	.301

CI = confidence interval, HR, hazard ratio.

### 3.4. The profile and prognostic role of dMMR status in CRC patients

Mismatch repair gene expression had an important role in CRC patients, especially in patients with 5-Fu treatment. We used the traditional IHC method to detect MMR status in our cohort. In Total, there were 46 CRC patients with dMMR status. As shown in Table 5, dMMR distribution was significantly associated with young and middle-aged patients (19.6%, *P* < .000), right-sided tumors (29.9%, *P* < .000), poorly differentiated carcinoma (35.1%, *P* < .000), and TNM stage II (19.5%, *P* = .010).

**Table 5:** Association of dMMR status and clinicopathologic features.

	MMR	
	dMMR (%)	*P*
Age (years)		**.000209**
≤60	33 (19.6)	
>60	13 (6.5)	
Sex		*.320*
Male	28 (11.3)	
Female	18 (14.9)	
Tumor site		**.0000000223**
Right	26 (29.9)	
Left	14 (10.7)	
Rectum	5 (3.4)	
Multisite tumors	1 (50.0)	
Histological type		**.006**
Adenocarcinoma	40 (11.9)	
Mucinous carcinoma	5 (20.0)	
Signet-ring cell carcinoma	1 (14.3)	
MANEC*	0 (0)	
Differentiation		**.000049**
Well	0 (0)	
Moderately	27 (9.4)	
Poorly	13 (35.1)	
Other/NA	6 (18.2)	
TNM stage		**.010**
TIS	0 (0)	
I	7 (12.5)	
II	29 (19.5)	
III	9 (7.2)	
IV	1 (2.8)	
Lymphovascular invasion		.220
Positive	9 (8.7)	
Negative	37 (14.0)	
Perineural invasion		.210
Positive	8 (8.6)	
Negative	(13.8)	

dMMR = deficient mismatch repair, MMR = mismatch repair, mut = mutation, pMMR = proficient mismatch repair, TIS = tumor in situ, WT = wild-type.

* Mixed adenoneuroendocrine carcinoma.

**Figure 2:**
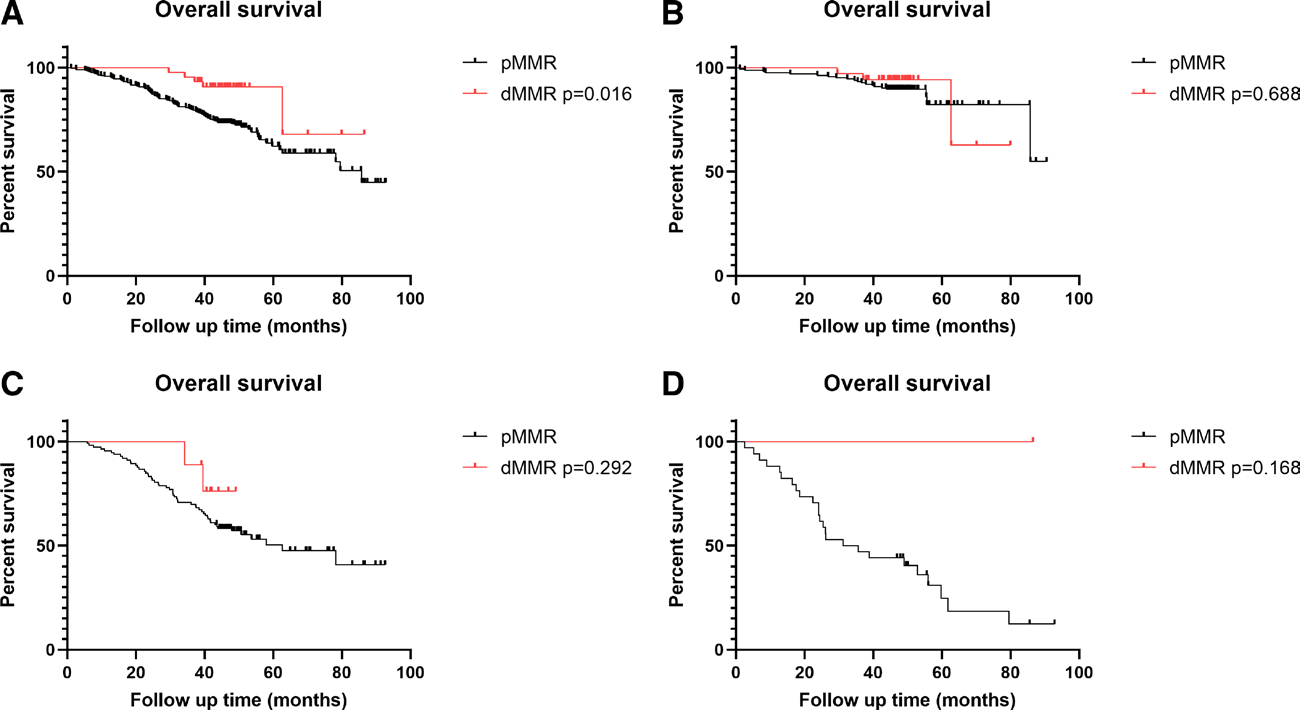
. dMMR status is associated with the prognosis in CRC patients. (A) Comparing the overall survival in all the CRC patients (*P* = .016). (B) Comparing the overall survival in CRC patients at the early stage (I–II) (*P* = .688). (C) Comparing the overall survival in CRC patients at stage III (*P* = .292). (D) Comparing the overall survival in CRC patients at stage IV (*P* = .0168). CRC = colorectal cancer, dMMR = deficient mismatch repair.

CRC patients with dMMR status had longer OS than patients with pMMR status (HR 0.348; 95% CI, 0.142–0.855, *P* = .021) in univariate analysis. However, multivariate analysis showed that MMR status was not an independent prognostic factor in CRC patients (HR, 0.507; 95% CI, 0.198–1.298, *P* = .156) (Table 6). In the Kaplan–Meier survival curves, the OS was shorter in all CRC patients with pMMR than in those with dMMR (*P* = .016) (Fig. 2A). When patients were stratified into different stages, dMMR status was not a prognostic factor in patients at an early stage (stage I–II) (Fig. 2B), or stage III or stage IV (Fig. 2C and D). In the Kaplan–Meier analysis of DFS, there was no significant difference in patients with pMMR or dMMR (*P* = .379) (Supplementary Figure S2, Supplemental Digital Content, http://links.lww.com/MD/I561).

**Table 6:** Univariate and multivariate analyses of prognostic impact of dMMR status.

Variables	Univariable analysis HR (95% CI)	*P*	Multivariable analysis HR (95% CI)	*P*
dMMR	0.348 (0.142–0.855)	**.021**	0.507 (0.198–1.298)	.156

CI = confidence interval, dMMR = deficient mismatch repair, HR = hazard ratio.

**Figure 3:**
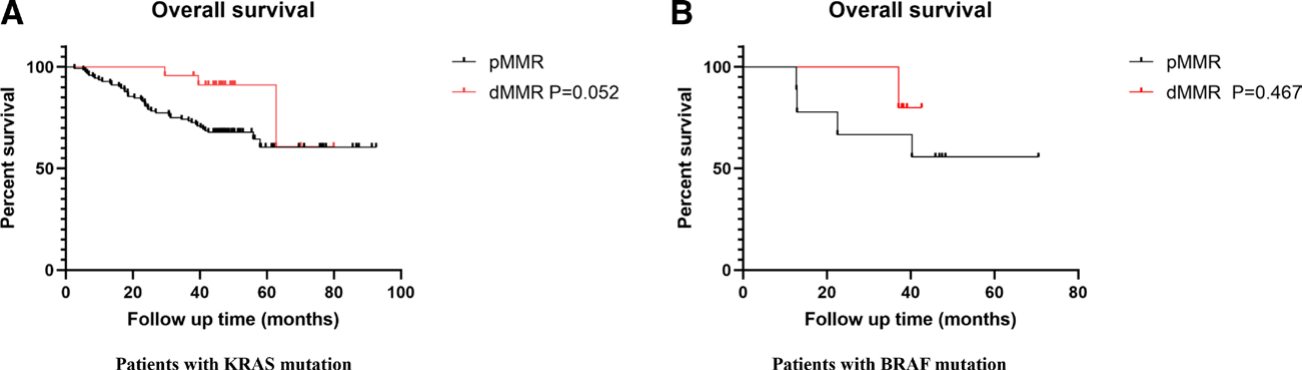
. dMMR status is not associated with the prognosis in CRC patients with KRAS or BRAF mutation. (A) Comparing the overall survival in CRC patients with KRAS mutation (*P* = .052). (B) Comparing the overall survival in CRC patients with BRAF mutation (*P* = .467). CRC = colorectal cancer, dMMR = deficient mismatch repair.

We further explored the role of the dMMR status in the driver gene mutation subgroup. Seven KRAS mutation cases were excluded because of a lack of follow-up information. As shown in Table 7, the percentage of patients with dMMR in the KRAS mutation group was slightly higher than that in the KRAS wild-type group (15.6% vs 10.5%, *P* = .103). However, the percentage of patients with dMMR in the BRAF mutation group was significantly higher than that in the BRAF wild-type group (35.7% vs 11.5%, *P* = .020). However, MMR status did not have significant power to predict the prognosis either in patients with KRAS mutations (*P* = .052) or BRAF mutations (*P* = .467) (Fig. 3A and B). The exact conclusion requires the inclusion of more patients with a longer follow-up period.

**Table 7:** Correlation of KRAS/BRAF mutation and MMR status in CRC.

	KRAS WT	KRAS Mut	*P*	BRAF WT	BRAF Mut	*P*
pMMR	187 (89.5%)	124 (84.4%)	.103	314 (88.2%)	9 (64.3%)	.020
dMMR	22 (10.5%)	23 (15.6%)		41 (11.5%)	5 (35.7%)	

CRC = colorectal cancer, dMMR = deficient mismatch repair, mut = mutation, pMMR = proficient mismatch repair, WT = wild-type.

## 4. Discussion

CRC is a common malignant epithelial neoplasm, and it occurs at a high rate in both men and women.^[14]^ The occurrence and progression of CRC are complex processes, and multiple alternative genetic pathways contribute to CRC development.^[15]^ As a result of improvements in surgical techniques and adjuvant and neoadjuvant therapies, the survival of patients with CRC is significantly prolonged. Therefore, precise treatment of long-survival CRC patients requires more detailed information about driver gene profiles. KRAS, NRAS, BRAF, and mismatch repair protein status are the main biomarkers used to stratify patients with CRC with different biomolecular characteristics. We reviewed the previous literatures with keywords “colon cancer,” “KRAS,” “NRAS,” and “BRAF,” and there was no agreed conclusion of the driver gene’s role in CRC patients. In our study, the frequencies of KRAS and BRAF mutations were similar to those previously reported; the frequency of NRAS mutations was slightly lower than that reported previously (1.6% vs 3–4%).^[20–24]^ The probable reason for this is that the NRAS mutation in our study did not include hotspots on exon 4. Our study was a single-center retrospective study that enrolled CRC patients who underwent surgery before 2018. Considering the detection efficiency and health economics, routine examination of driver gene mutation in our hospital covered the hotspots on Exon 2 to 3 of KRAS/NRAS and BRAF V600E. The correlations between KRAS mutations and clinicopathological characteristics in our study were consistent with other published studies showing that patients bearing KRAS mutations are associated with aggressive histological features such as advanced tumor status, poor differentiation, and distal metastasis.^[16]^ KRAS mutation was associated with right-sided colon tumor (62.1%, *P* < .000), and hotspot mutation on codon 12 exon 2 was dominant, accounting for 75.1% of all KRAS alleles (Tables 2 and 3). In our study, CRC patients with KRAS mutations had shorter OS than those with wild-type KRAS at stage IV (Fig. 1D). There are conflicting views regarding the prognostic role of KRAS mutations with specific point mutation. Some large multicenter cohorts have indicated that CRC patients bearing G12V or G12C had worse OS prognoses than wild-type KRAS tumors.^[17,18]^ However, KRAS mutation variants G12V and G12D had no impact on survival in univariate and multivariate analyses.^[19]^ We also did not observe difference in the impact of KRAS mutation variants G13D, G12D, or G12A on OS in our study (*P* = .705, *P* = .487, respectively) (Supplementary Figure S2, Supplemental Digital Content, http://links.lww.com/MD/I561 and Supplementary Figure S3, Supplemental Digital Content, http://links.lww.com/MD/I562). Recently a small molecular compound AMG 510, which inhibits RAS activity by directly targeting KRAS G12C, broke the dilemma of CRC patients bearing RAS mutations.^[16,20–22]^ KRAS G12C, a new star mutation, occurs in 3% of the CRC population in a published study.^[23]^ Our study indicated that the accurate frequency of KRAS G12C in China is slightly lower (2.2%) than in Western countries. However, it is still necessary to cover more hotspots of the RAS or BRAF genes as early as possible after surgery. Because genomic DNA degrades over the years, low-frequency mutations may not be detected. Previous research reported that KRAS and BRAF mutations were independent risk factors for shorter OS in Stage IV tumors (*P* = .038, *P* = .004, respectively), and NRAS mutation was an independent risk factor for shorter OS in Stage I to II tumors (*P* = .002). NRAS and BRAF V600E mutations had no effect on OS or DFS in our study (data not shown). One possible reason may be the small sample size, another possible reason is that we enrolled samples without bias.

Immunotherapy has received enormous interest in the treatment of many solid tumors, including CRC. In CRC, dMMR or MSI-H patients could benefit from immunotherapy and prolong OS. Some studies suggested that MMR status can guide the use of adjuvant chemotherapy in patients with early CRC; dMMR has a positive prognostic impact in patients with tumor low-risks.^[24,25]^ For example, a clinical trial (ClinicalTrials.gov, NCT03926338) in Sixth Affiliated Hospital, Sun Yat-sen University showed that 17 (88%) patients with dMMR or MSI-H CRC in the toripalimab plus celecoxib group and 11 (65%) patients with dMMR or MSI-H CRC in the toripalimab monotherapy group had a pathological complete response.^[26]^

Therefore, understanding the profile of dMMR or MSI-H in CRC patients and deciphering its association with clinicopathological characteristics is vital for neoadjuvant immunotherapy guidance. The percentage of dMMR cases was 12.5% in our cohort. dMMR status was more likely to occur in patients with CRC aged <60 years, right-sided tumors, poor differentiation, and TNM stage II (Table 5). These results are similar to previously published data.^[27–30]^ Most studies have discovered that dMMR status has a positive prognostic role for colorectal carcinoma in TNM.^[25]^ In our cohort, patients with dMMR status predicted longer OS not only in stage II but also in patients with other stages. However, multivariate analyses showed that the dMMR was not an independent prognostic factor for CRC. This may be due to the small number of advanced cancer patients in our study, most of whom underwent neoadjuvant therapy and were therefore excluded from the study.

Taieb et al discovered that KRAS mutations were independently associated with shorter OS in patients with MSS but not MSI.^[31]^ BRAF mutations are considered to have a poor prognosis, and several studies have suggested that the detrimental role of BRAF mutations is linked to microsatellite status.^[32,33]^ A retrospective analysis by Hendrik et al found that among 2000 patients with CRC, BRAF mutations and microsatellite status were associated with tumor stage; the proportion of MSS/BRAF mutant tumors increased with cancer stage (1.9% in stage I to 8% in stage IV), whereas the share of MSI/BRAF mutant tumors decreased with tumor stage (4% in stage I to 0.3% in stage IV).^[34]^ In our study, the percentage of dMMR status was higher in the BRAF mutation subgroup than in the BRAF wild-type group (35.7% vs 11.5%, *P* = .020). We analyzed the potential prognostic role of the pMMR status in patients with KRAS or BRAF mutations. There was no significant difference in OS between the pMMR and dMMR groups in the KRAS or BRAF mutation subgroup (*P* = .052, *P* = .467, respectively). The *P* value was close to 0.05; therefore, it is necessary to expand the sample size for further confirmation. The strong association between BRAF mutations and MSI could represent a new standard of treatment for this subgroup as well as a combination of chemotherapy, anti-vascular endothelial growth factor agents, and immune checkpoint inhibitors.

This was a retrospective single-center study, and the actual detections of gene mutations were performed nearly 10 years ago. Our study has unavoidable limitations. First, the sample size was not sufficient to make statistically significant conclusions. We observed some tendency in the prognostic role of KRAS and BRAF mutations, but solid conclusions need to be drawn from more data. Second, we tried to explore the role of dMMR status in patients with CRC, but the cases in our cohort lacked adjuvant therapy or neoadjuvant therapy information. Third, driver gene mutations were detected by the amplification refractory mutation system-polymerase chain reaction method, and it is difficult to report a low-frequency mutation of <1%. This may also be the reason for the relatively lower BRAF and NRAS mutations in our study compared to other published data. Although our study had these flaws, we provided real-world reality in a developing country, which is hardly implied by high-cost next-generation sequencing in routine examinations. Another dilemma in a developing country is that thousands of samples need to be tested for gene mutations every month. The amplification refractory mutation system-polymerase chain reaction method and proven kits which cover the definite hotspot mutation are much more suitable than large panel NGS in routine clinical settings.

In summary, our study provided the profile of KRAS NRAS and BRAF mutations in CRC and found that KRAS mutations are associated with inferior survival in patients with advanced CRC. dMMR status predicted longer OS in all CRC patients. BRAF mutation correlated with a higher percentage of dMMR status, indicating that patients with BRAF mutation and dMMR status would benefit from immunotherapy combined with targeted therapy.

## Author contributions

**Conceptualization:** Shen-Yi Lian, Ai-Wen Wu, Zhong-Wu Li

**Data curation:** Shen-Yi Lian, Lu-Xin Tan, Xin-Zhi Liu, Lu-Jing Yang, Ning-Ning Li, Li-Xin Zhou, Ting-Ting Sun, Lin Wang

**Investigation:** Lu-Xin Tan, Xin-Zhi Liu

**Methodology:** Ning-Ning Li, Qing Feng, Ping Wang, Yue Wang, Dong-Bo Qiao

**Supervision:** Ai-Wen Wu, Zhong-Wu Li

**Writing – original draft:** Shen-Yi Lian, Lu-Xin Tan

## Supplementary Material









## References

[1] Carethers JM Jung BH. Genetics and genetic biomarkers in sporadic colorectal cancer. Gastroenterology. 2015;149:1177–1190.e3. 26216840 10.1053/j.gastro.2015.06.047 PMC4589489

[2] Dekker E Tanis PJ Vleugels JLA. Colorectal cancer. Lancet. 2019;394:1467–80. 31631858 10.1016/S0140-6736(19)32319-0

[3] Ahmed M. Colon cancer: a clinician’s perspective in 2019. Gastroenterology Res. 2020;13:1–10. 32095167 10.14740/gr1239 PMC7011914

[4] Dienstmann R Vermeulen L Guinney J. Consensus molecular subtypes and the evolution of precision medicine in colorectal cancer. Nat Rev Cancer. 2017;17:79–92. 28050011 10.1038/nrc.2016.126

[5] Boland CR Thibodeau SN Hamilton SR. A National Cancer Institute Workshop on Microsatellite Instability for cancer detection and familial predisposition: development of international criteria for the determination of microsatellite instability in colorectal cancer. Cancer Res. 1998;58:5248–57. 9823339

[6] Diao Z Han Y Chen Y. The clinical utility of microsatellite instability in colorectal cancer. Crit Rev Oncol Hematol. 2021;157:103171. 33290824 10.1016/j.critrevonc.2020.103171

[7] Vilar E Gruber SB. Microsatellite instability in colorectal cancer-the stable evidence. Nat Rev Clin Oncol. 2010;7:153–62. 20142816 10.1038/nrclinonc.2009.237 PMC3427139

[8] Ganesh K Stadler ZK Cercek A. Immunotherapy in colorectal cancer: rationale, challenges and potential. Nat Rev Gastroenterol Hepatol. 2019;16:361–75. 30886395 10.1038/s41575-019-0126-x PMC7295073

[9] Samatar AA Poulikakos PI. Targeting RAS-ERK signalling in cancer: promises and challenges. Nat Rev Drug Discov. 2014;13:928–42. 25435214 10.1038/nrd4281

[10] Boutin AT Liao WT Wang M. Oncogenic KRAS drives invasion and maintains metastases in colorectal cancer. Genes Dev. 2017;31:370–82. 28289141 10.1101/gad.293449.116 PMC5358757

[11] Sanz-Garcia E Argiles G Elez E. BRAF mutant colorectal cancer: prognosis, treatment, and new perspectives. Ann Oncol. 2017;28:2648–57. 29045527 10.1093/annonc/mdx401

[12] Auclin E Zaanan A Vernerey D. Subgroups and prognostication in stage III colon cancer: future perspectives for adjuvant therapy. Ann Oncol. 2017;28:958–68. 28453690 10.1093/annonc/mdx030

[13] Stintzing S Heinemann V Moosmann N. The treatment of colorectal carcinoma with monoclonal antibodies: the importance of KRAS mutation analysis and EGFR status. Dtsch Arztebl Int. 2009;106:202–6. 19471640 10.3238/arztebl.2009.0202 PMC2680580

[14] Siegel RL Miller KD Fuchs HE. Cancer statistics, 2021. CA Cancer J Clin. 2021;71:7–33. 33433946 10.3322/caac.21654

[15] Yu L Huang S Lv W. [Research progress of the role of EMT in EGFR-TKIs resistance of non-small cell lung cancer]. Zhongguo fei ai za zhi = Chinese journal of lung cancer. 2018;21:907–11. 30591098 10.3779/j.issn.1009-3419.2018.12.08 PMC6318572

[16] Zhu G Pei L Xia H. Role of oncogenic KRAS in the prognosis, diagnosis and treatment of colorectal cancer. Mol Cancer. 2021;20:143. 34742312 10.1186/s12943-021-01441-4 PMC8571891

[17] Imamura Y Morikawa T Liao X. Specific mutations in KRAS codons 12 and 13, and patient prognosis in 1075 BRAF wild-type colorectal cancers. Clin Cancer Res. 2012;18:4753–63. 22753589 10.1158/1078-0432.CCR-11-3210 PMC3624899

[18] Jones RP Sutton PA Evans JP. Specific mutations in KRAS codon 12 are associated with worse overall survival in patients with advanced and recurrent colorectal cancer. Br J Cancer. 2017;116:923–9. 28208157 10.1038/bjc.2017.37 PMC5379149

[19] Modest DP Ricard I Heinemann V. Outcome according to KRAS-, NRAS- and BRAF-mutation as well as KRAS mutation variants: pooled analysis of five randomized trials in metastatic colorectal cancer by the AIO colorectal cancer study group. Ann Oncol. 2016;27:1746–53. 27358379 10.1093/annonc/mdw261 PMC4999563

[20] Karapetis CS Khambata-Ford S Jonker DJ. K-RAS mutations and benefit from cetuximab in advanced colorectal cancer. N Engl J Med. 2008;359:1757–65. 18946061 10.1056/NEJMoa0804385

[21] Stalnecker CA Der CJ. RAS, wanted dead or alive: advances in targeting RAS mutant cancers. Sci Signal. 2020;13:eaay6013. 32209699 10.1126/scisignal.aay6013 PMC7393681

[22] Amodio V Yaeger R Arcella P. EGFR blockade reverts resistance to KRAS(G12C) inhibition in colorectal cancer. Cancer Discov. 2020;10:1129–39. 32430388 10.1158/2159-8290.CD-20-0187 PMC7416460

[23] Hong DS Fakih MG Strickler JH. KRAS(G12C) inhibition with sotorasib in advanced solid tumors. N Engl J Med. 2020;383:1207–17. 32955176 10.1056/NEJMoa1917239 PMC7571518

[24] Gkekas I Novotny J Fabian P. Deficient mismatch repair as a prognostic marker in stage II colon cancer patients. Eur J Surg Oncol. 2019;45:1854–61. 31186203 10.1016/j.ejso.2019.05.023

[25] Merok MA Ahlquist T Røyrvik EC. Microsatellite instability has a positive prognostic impact on stage II colorectal cancer after complete resection: results from a large, consecutive Norwegian series. Ann Oncol. 2013;24:1274–82. 23235802 10.1093/annonc/mds614 PMC3629894

[26] Hu H Kang L Zhang J. Neoadjuvant PD-1 blockade with toripalimab, with or without celecoxib, in mismatch repair-deficient or microsatellite instability-high, locally advanced, colorectal cancer (PICC): a single-centre, parallel-group, non-comparative, randomised, phase 2 trial. Lancet Gastroenterol Hepatol. 2022;7:38–48. 34688374 10.1016/S2468-1253(21)00348-4

[27] Bae JM Kim JH Kang GH. Molecular subtypes of colorectal cancer and their clinicopathologic features, with an emphasis on the serrated neoplasia pathway. Arch Pathol Lab Med. 2016;140:406–12. 27128298 10.5858/arpa.2015-0310-RA

[28] Boland CR Goel A. Microsatellite instability in colorectal cancer. Gastroenterology. 2010;138:2073–2087.e3. 20420947 10.1053/j.gastro.2009.12.064 PMC3037515

[29] Lin CC Lai YL Lin TC. Clinicopathologic features and prognostic analysis of MSI-high colon cancer. Int J Colorectal Dis. 2012;27:277–86. 22076610 10.1007/s00384-011-1341-2

[30] Jang S Hong M Shin MK. KRAS and PIK3CA mutations in colorectal adenocarcinomas correlate with aggressive histological features and behavior. Hum Pathol. 2017;65:21–30. 28188750 10.1016/j.humpath.2017.01.010

[31] Taieb J Le Malicot K Shi Q. Prognostic value of BRAF and KRAS mutations in MSI and MSS stage III colon cancer. J Natl Cancer Inst. 2017;109:djw272. 28040692 10.1093/jnci/djw272 PMC6075212

[32] Roth AD Tejpar S Delorenzi M. Prognostic role of KRAS and BRAF in stage II and III resected colon cancer: results of the translational study on the PETACC-3, EORTC 40993, SAKK 60-00 trial. J Clin Oncol. 2010;28:466–74. 20008640 10.1200/JCO.2009.23.3452

[33] Taieb J Zaanan A Le Malicot K. Prognostic effect of BRAF and KRAS mutations in patients with stage III colon cancer treated with leucovorin, fluorouracil, and oxaliplatin with or without cetuximab: a post hoc analysis of the PETACC-8 trial. JAMA Oncol. 2016;2:643–53. 26768652 10.1001/jamaoncol.2015.5225

[34] Bläker H Alwers E Arnold A. The association between mutations in BRAF and colorectal cancer-specific survival depends on microsatellite status and tumor stage. Clin Gastroenterol Hepatol. 2019;17:455–462.e6. 29660527 10.1016/j.cgh.2018.04.015

