# Supplementary material for: KRAS, NRAS, BRAF signatures, and MMR status in colorectal cancer patients in North China
Source: Medicine (Baltimore). 2023 Mar 3;102(9):e33115. doi: 10.1097/MD.0000000000033115 (PMC9981427; doi:10.1097/MD.0000000000033115)
Supplement: Supplementary file 1 [file medi-102-e33115-s001.pdf]

Table S1 Univariate analysis of clinicopathological characteristics

Variables	Univariable analysis HR (95% CI)	*p*
**Age (＞60)**	1.515 (1.016-2.258)	**.041**
**Gender**		
Male	0.992(0.653-1.507)	.970
Female	1	
**Tumor Site**		
Right	1	
Left	0.605 (0.361-1.012)	.056
Rectum	0.819 (0.513-1.308)	.403
Multisite tumors	——	
**Histological type**		
Adenocarcinoma	1	
Mucinous carcinoma	1.361 (0.685-2.704)	.379
Signet-ring cell carcinoma	4.953 (2.001-12.261)	**.001**
Mixed adenoneuroendocrine carcinoma	18.024 (2.419-134.281)	**.005**
**Differentiation**		
Well	1	
Moderately	0.324 (0.149-0.707)	**.005**
Poorly	0.375 (0.142-0.989)	**.047**
Other/NA[a]	0.680 (0.276-1.673)	.401
**TNM stage**		
TIS	——	
I	0.120 (0.049-0.293)	< **.000**
II	0.125 (0.067-0.233)	< **.000**
III	0.540 (0.335-0.868)	**.011**
IV	1	
**Lymphovascular invasion positive**	2.585 (1.754-3.811)	< **.000**
**Perineural invasion positive**	2.426 (1.636-3.597)	< **.000**

TIS: Tumor In Situ

Table S2 Multivariate analysis of clinicopathological characteristics

Variables	Multivariable analysis HR (95% CI)	*p*
**Age (＞60)**	1.521 (1.001-2.311)	**.049**
**Histological type**		
Adenocarcinoma	1	
Mucinous carcinoma	0.152 (0.018-1.282)	.083
Signet-ring cell carcinoma	0.557 (0.061-5.069)	.604
**Differentiation**		
Well	1	
Moderately	0.167 (0.072-0.384)	< **.000**
Poorly	0.135 (0.048-0.381)	< **.000**
**TNM stage**		
Ⅰ	0.112 (0.044-0.290)	< **.000**
Ⅱ	0.123 (0.063-0.243)	< **.000**
Ⅲ	0.436 (0.264-0.722)	**.001**
Ⅳ	1	
**Lymphovascular invasion +**	1.164 (0.702-1.930)	.557
**Perineural invasion +**	1.480 (0.935-2.343)	.094
