# Supplementary material for: KRAS, NRAS, BRAF signatures, and MMR status in colorectal cancer patients in North China
Source: Medicine (Baltimore). 2023 Mar 3;102(9):e33115. doi: 10.1097/MD.0000000000033115 (PMC9981427; doi:10.1097/MD.0000000000033115)

Supplementary Figure 1 dMMR status is not associated with the prognosis in CRC patients with KRAS mutation (p=.224).

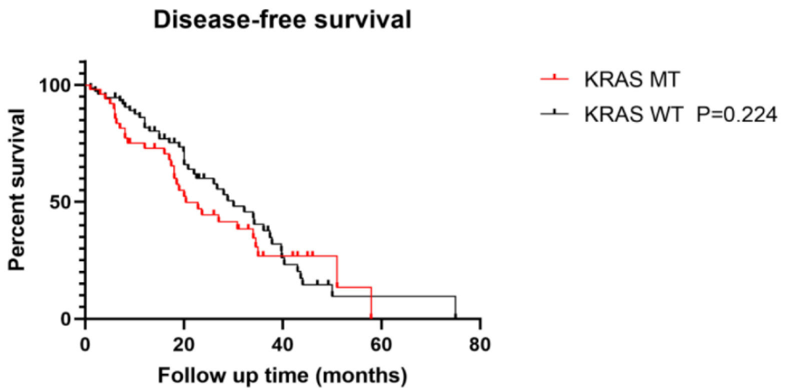

Supplement: Supplementary file 2 [file medi-102-e33115-s002.pdf]
